# Dynamics of an algae–bacteria microcosm: Photosynthesis, chemotaxis, and expulsion in inhomogeneous active matter

**DOI:** 10.1073/pnas.2410225122

**Published:** 2025-03-17

**Authors:** Praneet Prakash, Yasa Baig, François J. Peaudecerf, Raymond E. Goldstein

**Affiliations:** ^a^Department of Applied Mathematics and Theoretical Physics, Centre for Mathematical Sciences, University of Cambridge, Cambridge CB3 0WA, United Kingdom; ^b^Institute de Physique de Rennes, UMR 6251, Universite Rennes, Rennes F-35000, France

**Keywords:** active matter, chemotaxis, photosynthesis

## Abstract

In nature, there are significant relationships known between microorganisms from two kingdoms of life, as in the supply of vitamin B_12_ by bacteria to algae. Such interactions motivate general investigations into the spatiotemporal dynamics of metabolite exchanges. Here we study by experiment and theory a model system: a coculture of the bacterium *Bacillus subtilis*, an obligate aerobe that is chemotactic to oxygen, and a nonmotile mutant of the alga *Chlamydomonas reinhardtii*, which photosynthetically produces oxygen when illuminated. Strikingly, when a shaft of light illuminates a thin, initially uniform suspension of the two, the chemotactic influx of bacteria to the photosynthetically active region leads to expulsion of the algae from that area. We propose that this effect arises from advection by the inhomogeneous bacterial concentration. The resulting generalization of Fick’s law has been proposed in the context of chemotaxis and is mathematically related to the “turbulent pumping” in magnetohydrodynamics.

## Significance

Symbiotic relationships between photosynthetic algae and bacteria are known to be important in a variety of natural habitats, but the spatiotemporal aspects of such interactions are poorly understood. Here, we explore by experiment and theory a realization of these interactions in which chemotactic bacteria respond to the oxygen produced by immotile green algae through photosynthesis. When the illumination triggering the photosynthesis is a circular shaft of light we find the surprising result that the chemotactic influx of bacteria ultimately results in the expulsion of algae. In developing a mathematical model of this behavior, it is necessary to consider a generalization of Fick’s law of diffusion that incorporates the effects of an inhomogeneous bacterial concentration on the transport of algae.

In the early 1880s, the biologist Theodor Engelmann performed experiments that were perhaps the first to use bacteria as sensors (1–3). Several years prior, he made the first observation of bacterial chemotaxis toward oxygen, by showing that putrefactive bacteria would migrate toward the chloroplasts of the filamentous alga *Spirogyra*. He then determined the “action spectrum” of photosynthesis—the wavelength-dependent rate of photosynthetic activity—by passing sunlight through a prism and projecting the spectrum onto a filamentous green alga held in a chamber that contained those same bacteria, which gathered around the algae in proportion to the local oxygen concentration, providing a direct readout of the oxygen production rate.

Although Engelmann’s system was engineered for a particular purpose, and at first glance involves a one-way exchange of oxygen for the benefit of bacteria, there are many examples of mutualistic exchanges between microorganisms from two kingdoms of life. One of considerable significance is that involving vitamin B_12_. In a landmark study (4), it was shown that a significant fraction of green algae that require this vitamin for their metabolism do not produce it, and as the ambient concentration of B_12_ in the aqueous environment is so low, they instead acquire it from a mutualistic relationship with bacteria, which benefit from carbon source.

The study of B_12_ transfer raises fascinating questions in biological physics related to the interplay of metabolite production, chemotaxis, and growth (5), including the issue of how organisms find each other (6) and stay together in the turbulent environment of the ocean, and how advection by fluid flows arising from microorganism motility affects such mutualisms. As it is difficult to control the production of vitamin B_12_, we sought to construct a system in which the production of a chemical species needed by one member of an interacting pair of organisms could be controlled by the experimentalist. Taking motivation both from Engelmann’s experiments and the B_12_ system, we introduce here a coculture (7) in which an obligate aerobic bacterium (one that requires oxygen) that is chemotactic toward oxygen coexists with a green alga whose photosynthetic activity can be turned on and off simply by controlling the external illumination. We use the bacterium *Bacillus subtilis*, whose aerotaxis (8, 9) has been central in the study of bioconvection (10) and in the discovery of “bacterial turbulence” (11), the dynamical state of a concentrated suspension with transient, recurring vortices and jets of collective swimming on scales large compared to the individual bacteria. The alga species is the well-studied unicellular *Chlamydomonas reinhardtii*, a model organism for biological fluid dynamics (12) with readily available motility mutants useful in probing the role of swimming in metabolite transfer. Together these define what we term the algae-bacteria-chemoattractant system (ABC). While coupled population dynamics problems have been studied from the familiar reaction–diffusion–chemotaxis point of view in bacterial range expansion (13), marine (14) and more general ecological contexts (15), a significant body of work has explored the dynamics of mixtures of passive and active particles in which complex dynamics may arise from the (fluid) dynamical features of the motility of the active component. Among the first effects to be studied was the way in which collective motion can act as a “thermal bath” in enhancing the diffusivity of suspended particles (16, 17), which in turn raises fundamental issues concerning generalizations of Fick’s law (18, 19), particularly in the presence of hydrodynamic shear (20). More recent studies have found that gradients of activity of the motile species can induce spatial segregation of the passive component, in contexts such as wall-induced circle-swimming of bacteria (21), formation of crystallites of passive microspheres due to phoretic (22) and other types of light-switchable phenomena associated with Janus particles (23, 24), as well as microfluidic confinement (25). Beyond synthetic systems, the implications of stochastically induced transport have been found to extend to biological cells, where they play a crucial role in dictating the positional distribution of cell organelles (26, 27). In addressing our experimental observations, the ABC model developed here encodes the effects of inhomogeneities in activity on the transport of passive particles through a mathematical formulation that goes beyond those utilized in the systems above and in prior work on chemotaxis in mixtures of particles (28).

**Fig. 1.**
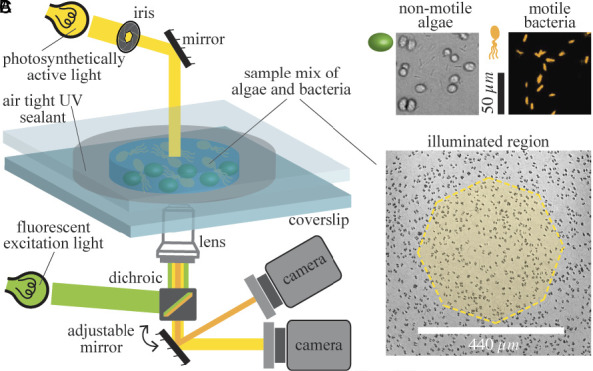
Experimental setup. (*A*) Schematic of system to illuminate a coculture of a fluorescent strain of the bacterium *B. subtilis* and a flagella-less mutant of the green alga *C. reinhardtii*, initially distributed uniformly within the sample chamber of depth 300 μm. After a shaft of visible light with a power of 20 μW (131.5 W$/m^{2}$) illuminates the central region, the algae produce oxygen to which the bacteria are attracted. For fluorescent imaging dichroic remains in the path of excitation light while for brightfield imaging dichroic is disengaged. (*B*) Algae and bacteria as observed through the brightfield and fluorescent channels, respectively. (*C*) Yellow shading indicates the illuminated region of the sample chamber, with dark algae visible in the background.

**Fig. 2.**
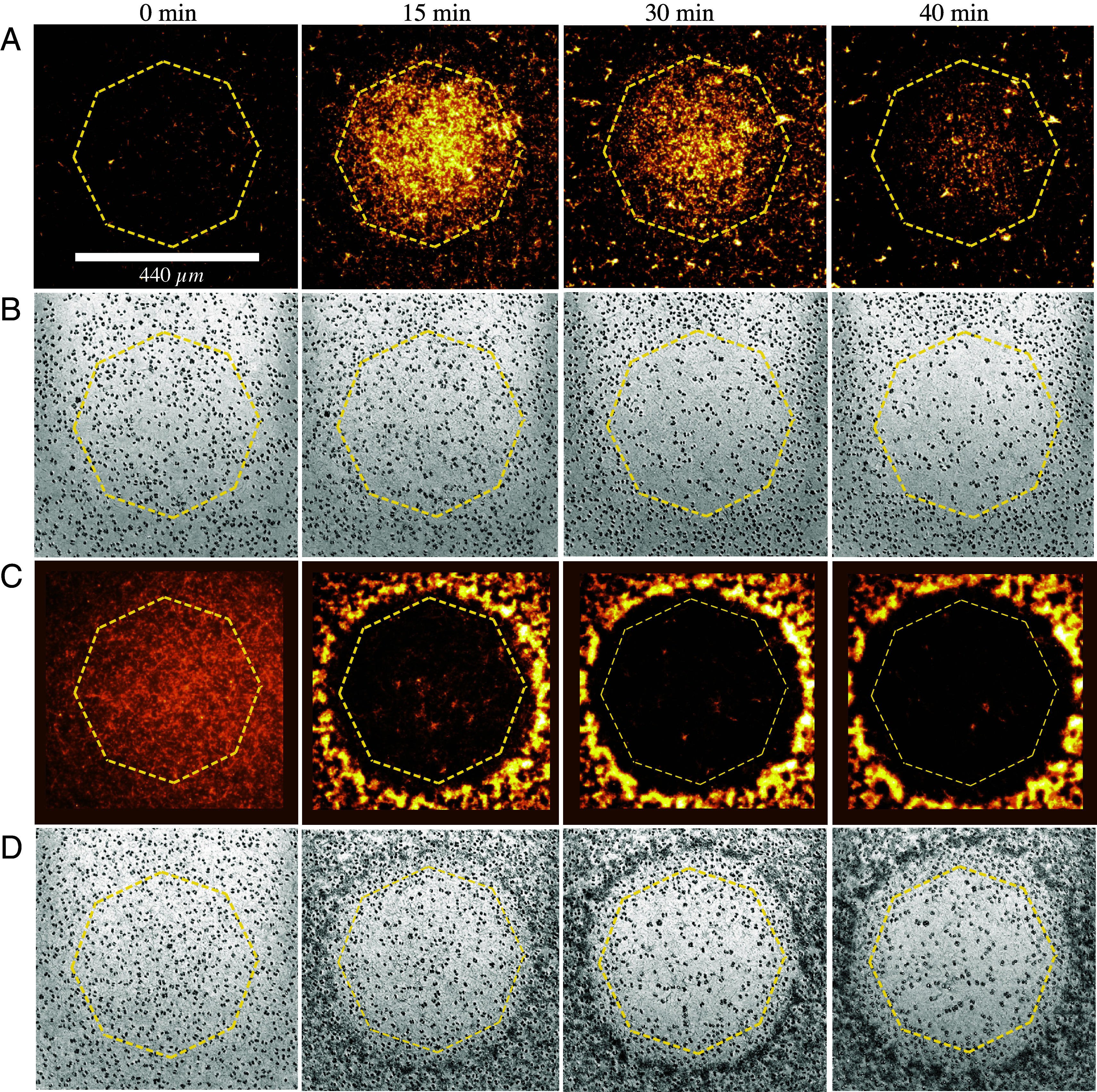
Bacterial influx and algal expulsion. See *SI Appendix*, Fig. S2 for additional examples of expulsion. (*A*) Type I dynamics. Spatiotemporal evolution of bacterial concentration after illumination is initiated, where octagonal ring indicates boundary of illuminated region. At the low initial bacteria concentration (∼1 × 10^8^ cm^−3^), bacteria first move into the illuminated region and then retreat. (*B*) Algal concentration as a function of time, demonstrating expulsion from illuminated region. (*C*) Type II dynamics. At a higher concentration (∼5 × 10^8^ cm^−3^), many bacteria become nonmotile and are expelled into the dark region, forming a concentrated circular accumulation that acts as a natural boundary. (*D*) Algae are also expelled, accumulating inside the confines of the bacterial accumulation.

Our experiment to understand the dynamics of the ABC system involves a thin, quasi-two-dimensional suspension of nonmotile algae and fluorescently labeled bacteria at initially uniform concentrations. As depicted in Fig. 1, a shaft of photosynthetically active light is cast on the suspension, triggering oxygen production by the illuminated algae. As shown in Fig. 2 *A* and *B*, this leads to chemotaxis of bacteria into the illuminated region, producing a high concentration of bacteria. Remarkably, we find that algae are then expelled from the illuminated region (see also Movie S1). Quantitative measurements of the local bacterial dynamics in the system show that this expulsion is associated with a gradient of bacterial concentration from its peak at the center, leading to an outward algal transport. On longer time scales, after the algal expulsion, the bacterial concentration returns to uniformity as the bacteria diffuse outward in the absence of chemotactic stimulus. We name this Type I dynamics. Fig. 2 *C* and *D* shows that at sufficiently high initial bacterial concentrations, a new, Type II behavior is observed; expulsion of algae and consumption of oxygen are sufficiently rapid that many bacteria in the illuminated region become hypoxic, transition to an immotile state, and are then also expelled into the dark (Movie S2).

We develop here a system of coupled PDEs for the dynamics of the ABC system that provides a quantitative account of the experimental observations. These PDEs incorporate diffusion, chemotaxis, oxygen production and consumption, and algal transport by stochastic bacterial motion. The expulsion of algae has interesting similarities to the process known in magnetohydrodynamics (MHD) as turbulent pumping, a mechanism of transport related to the diamagnetic effect of turbulence first described by Zel’dovich (29) in which gradients of turbulent intensity lead to transport of magnetic flux (30–32). We show here that inclusion of advective contributions to the algal dynamics leads to a mathematical structure identical to that found in the turbulent pumping problem.

## Results

**Fig. 3.**
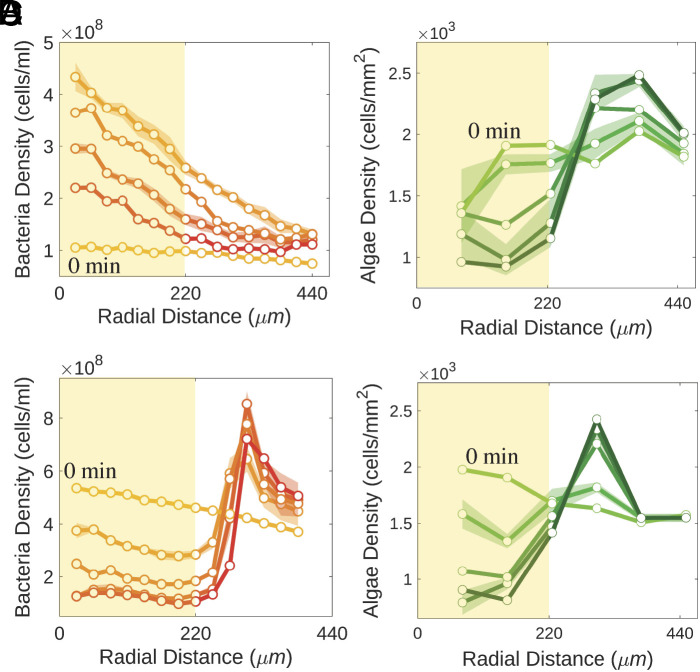
Bacterial accumulation and algal expulsion. Yellow shading in the plots up to 220μm indicates illuminated region. SE evaluated over the four quadrants is shown for 10 and 30 min intervals. (*A*) Bacterial and (*B*) algal concentrations for a coculture with initial concentrations $b=1\times 10^{8}$ cm^−3^ and $a=5\times 10^{8}$ cm^−3^, showing Type I dynamics. Shading of symbols and lines increase with time, with a 10 min interval between datasets. (*C* and *D*) As in (*A* and *B*) but with initial bacterial concentration $b=5\times 10^{8}$ cm^−3^, exhibiting Type II dynamics.

Figs. 2 and 3 (along with Movies S1 and S2) summarize the main experimental observations associated with a homogeneous initial condition in darkness that is then illuminated with a shaft of light. The concentration of algae was fixed at $5\times 10^{6}$ cm^−3^ while varying the bacteria concentration in the range $\left(1\mathrm{to}5\right)\times 10^{8}$ cm^−3^. For an initial bacterial concentration of $b=1\times 10^{8}$cm^−3^ giving Type I dynamics (Movie S1), we observe over the course of the first ∼10 min after the start of illumination that the concentration of bacteria at the center of the illuminated region increases dramatically, as visualized in Fig. 2*A* and quantified in Fig. 3*A*, reaching a peak enhancement of a factor of ∼5, with a roughly linear decrease out to the edge. That peak then relaxes away until the bacterial concentration is again nearly uniform after ∼35 min. At the peak of accumulation, after ∼15 min, there is a clear bacterial depletion zone just outside the illuminated region, extending to a radius of ∼400 to 500 μm from the center of the illuminated region. During this period, as shown in Figs. 2*B* and 3*B*, the algal concentration becomes strongly depleted within the illuminated region, with accumulation at its boundary.

**Fig. 4.**
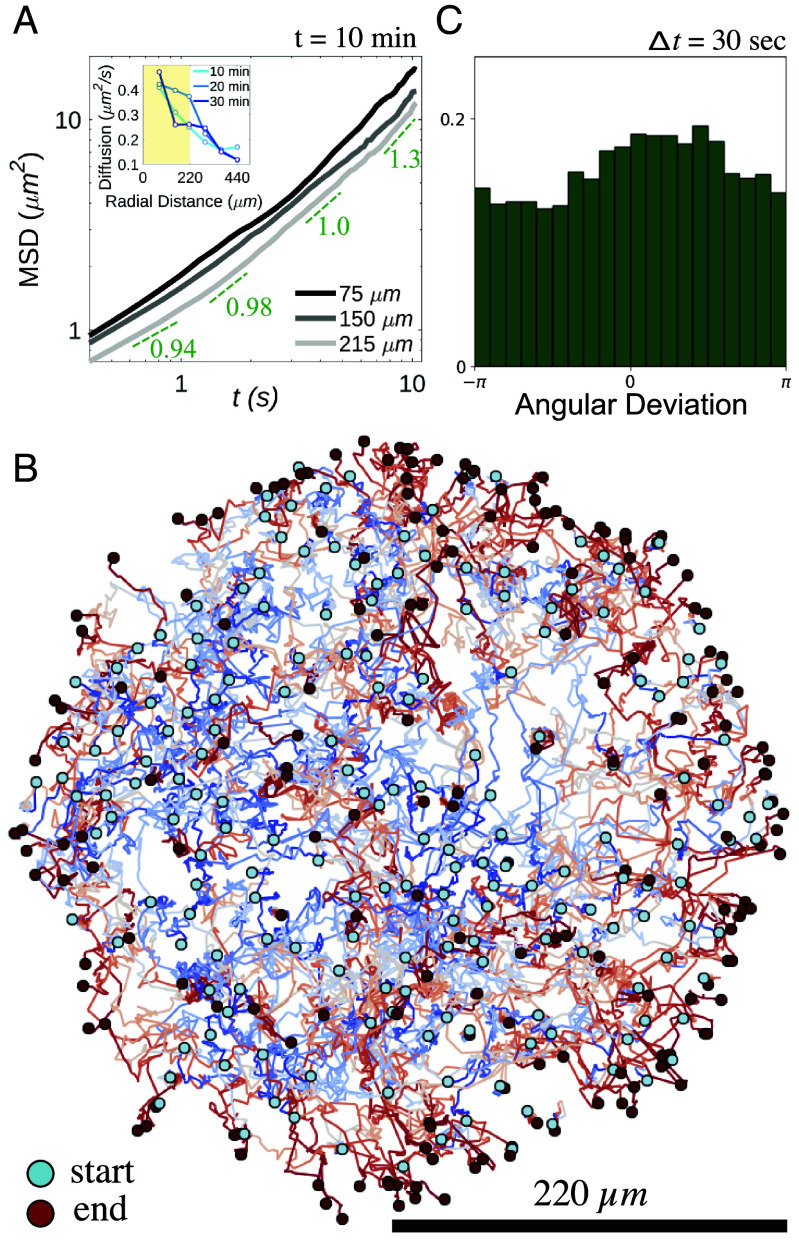
Algal dynamics during expulsion. (*A*) MSD of algae versus time for the cases shown in Fig. 3 *A* and *B* at different radii of width 75μm in the illuminated region. Dashed lines indicate apparent slope at different times. *Inset* shows algal diffusivity determined in linear regime of MSD versus radial distance at various times after start of illumination. (*B*) Algal trajectories for Type I dynamics. Coloring along trajectories runs from blue (initial) to red (final) as algae are expelled from illuminated region. (*C*) Probability distribution of algal displacements for Δt=30s as a function of angle with respect to the outward normal from the domain, illustrating peak in the outward direction.

We measured the mean squared displacement (MSD) of algae versus time at a range of radii *r* from the light shaft center. The MSD in Fig. 4*A* exhibits systematic upward curvature, with a local exponent of unity at time $t_{1}∼$6 to 7 s, but faster behavior for $t>t_{1}$. Such superdiffusion for tracers is a well-known consequence of collective behavior in concentrated bacterial suspensions (11, 16). A heuristic illustration of the strong gradient in collective behavior in the illuminated region is obtained by determining an effective algal diffusion constant ${\tilde{D}}_{a}$ from the slope of the MSD curve at *t*_1_ (*Inset* of Fig. 4*A*), which is a strongly decreasing function of distance from the shaft center, that mirrors the decreasing bacterial concentration. The largest of these diffusivities is ∼17 times the purely thermal value $D_{\mathrm{th}}=k_{B}T/6\pi \mu a≃0.03\mu$m^2^/s, where *μ* is the medium viscosity and a=6.5μm is twice the algal radius since most algae exist as pairs (“palmelloids”). Yet, by itself, an effective algal diffusivity ${\tilde{D}}_{a}∼0.5\mu$m^2^/s implies a diffusive time $R^{2}/{\tilde{D}}_{a}∼1,600$min for algae at the center to escape purely by random motion, far longer than the observed time of 30min; the superdiffusive behavior seen for $t>t_{1}$ in Fig. 4*A* signals a distinct transport process. This conclusion is supported by capturing the trajectories of every algal cell within the illuminated region during the entire expulsion process, shown overlaid in Fig. 4*B* and in Movie S3. These trajectories are obtained at a sampling rate of 1 frame/s (fps). Subsampling them at larger time intervals gives insight into the angular distribution of displacements associated with interactions between bacteria and algae, as in the original notion of bacteria as a “bath” of particles analogous to the solvent molecules whose collisions with a tracer particle give rise to ordinary Brownian motion. Fig. 4*C* shows those displacements for the time interval Δt=30s, expressed as a function of the angle with respect to the outward normal from the domain at the location of each alga. We see a clear azimuthal nonuniformity with a peak in the outward direction. We infer that the expulsion process is driven by this inhomogeneous stochastic forcing, itself arising from the local gradient of bacteria created by their chemotaxis.

At the higher initial bacterial concentration $b=5\times 10^{8}$cm^−3^ we observe the distinct Type II behavior (Movie S2), as seen in Figs. 2 *C* and *D* and 3 *C* and *D*: expulsion happens much more rapidly, the bacterial concentration profile becomes nonmonotonic in radius, with a strong peak just outside the illuminated region, and the peak of the concentration of expelled algae is narrower. Closer microscopic inspection of the region of high bacteria concentration in the dark region shows (i) that most of the bacteria there are immotile and (ii) that the expelled algae reside just inside the ring of expelled bacteria. We deduce that many bacteria in the illuminated region have become hypoxic and are expelled from that area through much the same process that expelled the algae. The algae in turn are confined by the ring of nonmotile bacteria.

### The ABC Model.

We now turn to a mathematical model for the behavior described above, focusing first on Type I expulsion. In this case, a minimal description of the system entails the concentration fields a(r,t),b(r,t) and c(r,t) for algae, bacteria, and oxygen, respectively. If *D*_*c*_ is the oxygen diffusion constant (assumed independent of all other variables), *k*_+_ is the rate of oxygen production per algal cell when illuminated with a spatially varying light intensity I(r) and the oxygen consumption rate per bacterium has a Michaelis–Menten form (33), then

[1] $$\frac{\partial c}{\partial t}=D_{c}\nabla^{2}c+k_{+}aI\left(r\right)-k_{-}b\frac{c}{K_{c}+c},$$

where *k*_−_ is the maximum consumption rate per cell and *K*_*c*_ is the Michaelis constant.

Consider first the situation of uniform illumination (*I* = 1) and uniform concentrations *a*_0_ and *b*_0_ of algae and bacteria. A steady state $c^{*}=K_{c}k/\left(1-k\right)$, with $k=k_{+}a_{0}/k_{-}b_{0}$, can be reached in which consumption balances production, provided *k* < 1. We assume this inequality is always satisfied and typically invoke the weak production limit *k* ≪ 1, in which the oxygen consumption can be approximated as $k_{-}bc/K_{c}$.

We explain algal expulsion from the illuminated region through three intermediate calculations; oxygen production in a uniform suspension; bacterial chemotaxis in the presence of oxygen production; algal dynamics due to an inhomogeneous bacterial concentration. Suppose that the light is constrained to a shaft of radius *R*. Scaling time, space, and concentrations via $T=tD_{c}/R^{2}$, η=r/R, $\chi =c/c^{*}$, $\alpha =a/a_{0}$, and $\beta =b/b_{0}$, letting ϵ=k/(1−k), and introducing the screening length

[2] $$\lambda ={\left(D_{c}\tau_{c}\right)}^{1/2},$$

where $\tau_{c}=K_{c}/k_{-}b_{0}$ is the characteristic consumption time of oxygen, the dynamics Eq. **1** takes the form

[3] $$\frac{\partial \chi }{\partial T}=\nabla^{2}\chi +\left(1-k\right)\kappa^{2}\alpha \Theta \left(1-\eta \right)-\kappa^{2}\beta \frac{\chi }{1+\epsilon \chi },$$

where now $\nabla^{2}=\nabla_{\eta }^{2}$, Θ is the Heaviside function, $\kappa^{2}=R^{2}/\lambda^{2}=\tau_{D}/\tau_{c}$, where $\tau_{D}=R^{2}/D_{c}∼25$ s is the time for oxygen to equilibrate diffusively across the illuminated region.

If we clamp concentrations *α* and *β* at unity, take *k* ≪ 1, and enforce continuity in *χ* and *χ*_*η*_ at *η* = 1, the steady state of Eq. **3** in an unbounded domain is

[4] $$\begin{array}{c} \chi \left(\eta \right)=\left\{\begin{array}{cc} 1-\kappa K_{1}\left(\kappa \right)I_{0}\left(\kappa \eta \right) & \eta \leq 1,\\ \kappa I_{1}\left(\kappa \right)K_{0}\left(\kappa \eta \right) & \eta \geq 1, \end{array}\right. \end{array}$$

in terms of modified Bessel functions *K*_*ν*_ and *I*_*ν*_. From the inner solution, we see that the oxygen concentration at the center of the illuminated region asymptotes to unity ($c^{*}$ in unrescaled units) for large domain size *κ* but is attenuated strongly as *κ* falls below unity. The outer solution behaves as χ∼ exp (−(r−R)/λ), showing that *λ* is the penetration depth of oxygen into the surrounding bacterial population and sets the rough scale of the depletion zone seen in Fig. 2*A*, which will be modified by chemotaxis (see below). With $D_{c}∼2\times 10^{3}\;\mu$m^2^/s and R=220 μm, and the estimate $\tau_{c}∼250$ s (34), we find λ∼700 μm, and thus *κ* ∼ 0.3.

Relaxing the assumption of uniform bacterial concentration, after a time *τ*_*D*_ bacteria within a distance *λ* of the edge of the illuminated region will experience the steepest oxygen gradient and chemotax most rapidly inward. This can be described by the simplest combination of diffusion and chemotaxis as in the Keller–Segel model (35), $\partial b/\partial t=D_{b}\nabla^{2}b-\nabla \cdot \left(gb\nabla c\right)$

[5] $$\frac{\partial \beta }{\partial T}=d\nabla^{2}\beta -\gamma \nabla \cdot \left(\beta \nabla \chi \right),$$

where $d=D_{b}/D_{c}$, and $\gamma =gc^{*}/D_{c}$.

A steady state can be reached when the chemotactic flux γβ∇χ balances the diffusive flux d∇β, yielding

[6] $$\beta_{\mathrm{ss}}\left(\eta \right)=A\exp\left(\frac{\gamma }{d}\chi \left(\eta \right)\right),$$

where *A* is a normalization constant. While this profile captures the observation that the bacterial concentration reaches its maximum at *η* = 0, where *χ* is maximized, the profile Eq. **6** only develops on time scales sufficient for bacteria outside the depletion zone to move inward and replenish partially the depletion. This time will be at least ${\left(R+\lambda \right)}^{2}/D_{b}\gg \tau_{D}$. Prior to this, the bacterial concentration is nonmonotonic, with the depletion zone seen in Fig. 2*A* being considerably smaller than the length *λ*.

The flagella-less algae used in our experiments do not swim; their movement arises from collective flows driven by the concentrated bacteria. At the very least this leads to enhanced diffusivity, which, as seen in Fig. 3*F*, varies in space, and by itself, is captured by an algal flux $J_{D}=-{\tilde{D}}_{a}\nabla a$. While this contribution is surely present, our observations of outward flux of alga from an initially uniform concentration suggest the growth of an unstable concentration mode during the depletion of algae, a phenomenon not captured by a purely diffusive term.

In proposing a second contribution to the algal flux, we note that the algae will be advected by the local flows created by the swimming of the bacteria (16, 17, 36) and will be reoriented by collisions with them, where both effects will vary in space due to the gradients of bacterial concentration. In quantifying these effects we take motivation from classic work (18) on run-and-tumble locomotion in chemotaxis which considered situations in which the local swimming speed ν(x) and tumble rate ω(x) for an organism may vary with position x. When the two are constant the organism density *σ* evolves via diffusion with a Fick’s law flux J=−D∇σ, with a diffusion constant $D\propto \nu^{2}/\omega$. In the general case that both *ν* and *ω* vary there is an additional contribution to the flux proportional to σ(ν/ω)∇ν. Carrying this over to the algal dynamics, we suggest that as collisions between swimming bacteria and immotile algae are ultimately responsible for transport of algae, gradients in bacterial concentration will lead to gradients of the effective speed *ν* of the algae. Assuming the simplest linear scalings *ν* ∝ *b* and *ω* ∝ *b*, we obtain a contribution to the algal flux J=−pa∇b for some *p* > 0. With the concentration seen in Fig. 3*A*, whose radial gradient is negative, this contribution leads to an outward flux of algae.

Assembling these contributions and rescaling as above, we obtain two equivalent forms of the dynamics,

[7a] $$\frac{\partial \alpha }{\partial T}=\nabla \cdot \left(d_{a}\nabla \alpha \right)+\zeta \nabla \cdot \left(\alpha \nabla \beta \right),$$

[7b] $$\frac{\partial \alpha }{\partial T}-\zeta \nabla \beta \cdot \nabla \alpha =\nabla \cdot \left(d_{a}\nabla \alpha \right)+\zeta \alpha \nabla^{2}\beta ,$$

where $d_{a}={\tilde{D}}_{a}/D_{c}$ and $\zeta =pb_{0}/D_{c}$. The first form Eq. **7a** shows a parallel to the bacterial chemotaxis equation Eq. **5**; algae exhibit negative “bacteria-taxis.” In the second form Eq. **7b**, we see that there is an explicit advective contribution and a “reactive” term $\alpha \nabla^{2}\beta$. The latter is the sought-after linearly destabilizing term for a homogeneous initial algal concentration. With $\nabla^{2}b<0$ inside the illuminated region, if *ζ* > 0 this term decreases the algal concentration *α* there, while the advective contribution moves alga outward.

**Fig. 5.**
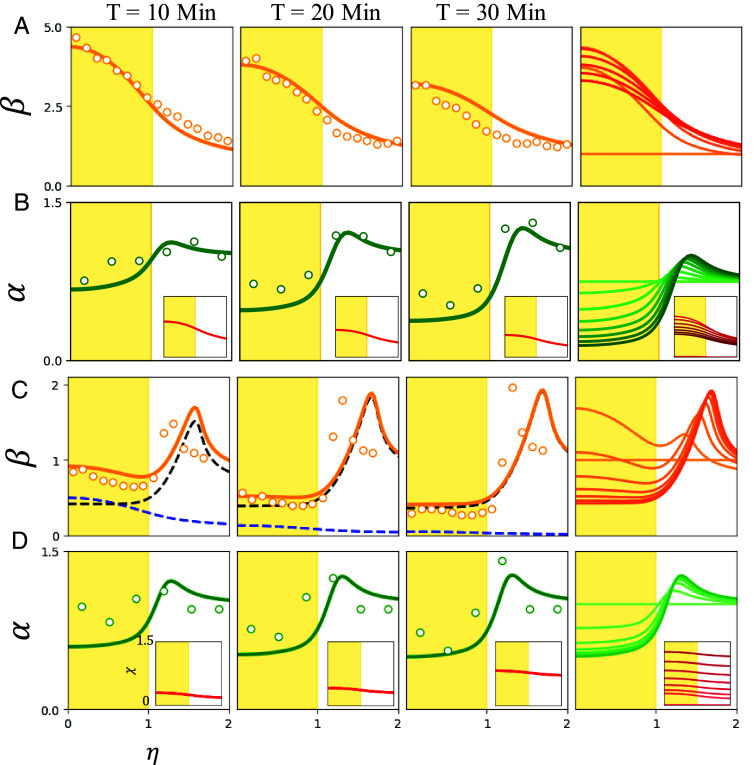
Theoretical predictions. (*A* and *B*) Type I dynamics. (*A*) Nondimensional bacterial dynamics from experiment (circles) compared to theoretical fit (solid lines) for first 30 min of experiment. The rightmost column in each row shows the theoretical time evolution over 30 min, sampled every ∼3 min. Shading intensity increases with time. (*B*) As in (*A*) but for algae, showing expulsion. *Insets* show theoretical oxygen profiles *χ*. (*C* and *D*) Type II dynamics. (*C*) Experimental bacterial dynamics (circles) compared to predictions of the ABCD model. Dashed lines indicate immotile (black) and active (blue) bacteria, whose sum is orange solid line. The rightmost plot shows time evolution of the total bacterial concentration. (*D*) Algal expulsion for high-density experiment. For (*A*) and (*B*), we took $\kappa^{2}=0.09,\epsilon =0.1,d_{b}=0.1,d_{a}=0.0001,\gamma =5.0,\zeta =0.002$. For (*C*) and (*D*), we use $\gamma =1.1,\zeta =0.01,\kappa^{2}=0.45,\epsilon =0.02,d_{\delta }=10^{-4},\rho =0.08,\chi^{*}=1.0,\zeta_{d}=0.075,\delta^{*}=0.01$ with all other parameter values held over.

The model defined by Eqs. **3**, **5**, and **7b** was solved numerically using standard pseudospectral spatial discretization and semi-implicit time marching on a 200 by 200 point grid with periodic boundary conditions and a domain size five times the radius of the illuminated region. *SI Appendix*, Table S3 details the values and sources (whether fixed or fitted) of the parameters used in these numerical studies. The initial conditions for numerical studies were taken to be *α* = 1, *β* = 1, *δ* = 0, and *χ* ≈ 0, where at *T* = 0, the light is switched on. Fig. 5 *A* and *B* shows how the model provides a quantitative fit to Type I dynamics. In particular, we see the prompt accumulation of bacteria in the illuminated region followed by expulsion of algae. In the model and in experiment, the fraction of algae ultimately expelled is ∼0.5, so there is still oxygen production within the illuminated region after ∼30 min, leading to continued chemotactic attraction of bacteria inward. This leads to a slower return of the bacterial concentration to its original value than seen in experiment. This may reflect processes such as bacterial adaptation to the oxygen and a gradual reduction in oxygen production by algae.

### The ABCD Model.

While the ABC model can account for the essential features of Type I dynamics, it does not allow for loss of motility of bacteria at low oxygen concentrations in Type II dynamics. This transformation has been discussed in the context of bioconvection (34, 37), where influx of oxygen at the air–water interface of a bacterial suspension competes with consumption within the fluid, leading to a hypoxic region hundreds of microns below the surface. Hypoxia-induced motility transitions have also been observed in the penetration of oxygen into suspensions of *Escherichia coli* (38).

To account for this transformation, we view the “dormant,” nonmotile state of the bacteria, with concentration *d*, as a separate population distinct from the motile form, so the extended “ABCD” model involves algae, bacteria, chemoattractant, and dormant bacteria. The interconversion rate as a function of oxygen concentration *c* is taken as a simple generalization of the substrate-dependent growth of the Monod model (39), $v_{\mathrm{con}}bK_{\mathrm{sat}}/\left(c+K_{\mathrm{sat}}\right)$, where *v*_*con*_ is the maximum conversion rate to the immotile form and *K*_*sat*_ is the concentration at which half-maximal conversion occurs.

The dynamics of dormant bacteria include generation, expulsion, and a very small diffusion constant *D*_*d*_. Setting $\delta =d/b_{0}$, the rescaled dynamics takes the form

[8] $$\frac{\partial \delta }{\partial T}-\zeta_{d}\nabla \beta \cdot \nabla \delta =d_{d}\nabla^{2}\delta +\zeta_{d}\delta \nabla^{2}\beta +\rho \beta \frac{\chi^{*}}{\chi^{*}+\chi },$$

where $d_{d}=D_{d}/D_{c}$, $\rho =v_{\mathrm{con}}R^{2}/D_{c}$ and $\chi^{*}=K_{\mathrm{sat}}/c^{*}$. Accordingly the bacterial dynamics Eq. **5** acquires the corresponding loss term from conversion, becoming

[9] $$\frac{\partial \beta }{\partial T}=d\nabla^{2}\beta -\gamma \nabla \cdot \left(\beta \nabla \chi \right)-\rho \beta \frac{\chi^{*}}{\chi^{*}+\chi }.$$

As we do not observe any conversion of dormant cells back to motile ones, the dynamics in Eqs. **8** and **9** involves only one-way conversion from motile to dormant bacteria. A final modification to the ABC model involves steric effects on algal diffusion that occur when the concentration of dormant bacteria is large. This is modeled by modifying the rescaled algal diffusivity *d*_*a*_ to $d_{a}\left(1-\delta /\left(\delta +\delta^{*}\right)\right)$, a form that reduces *d*_*a*_ at large *δ* but retains its positivity. In numerical studies, we take the initial conditions to be α=1,β=1,δ=0, and *χ* ≈ 0 everywhere so that originally only motile bacteria are present. This is consistent with observation of swimming bacteria present throughout our domain at the start of the experiment. With these components, Fig. 5 *C* and *D* show that the ABCD model captures the expulsion of both algae and immotile bacteria, with a peaked accumulation of dormant bacteria blocking transport of algae. Our numerical studies (*SI Appendix*, Fig. S4) show that there is a smooth variation in the degree of algal and dormant bacterial expulsion as a function of the initial concentration of bacteria. In this simplest model of phenotypic switching, we have not included any respiration by the dormant bacteria; doing so would provide a sink for oxygen outside the illuminated region, reducing the slow oxygen accumulation otherwise present, as seen in Fig. 5.

### Spatially Averaged Model.

**Fig. 6.**
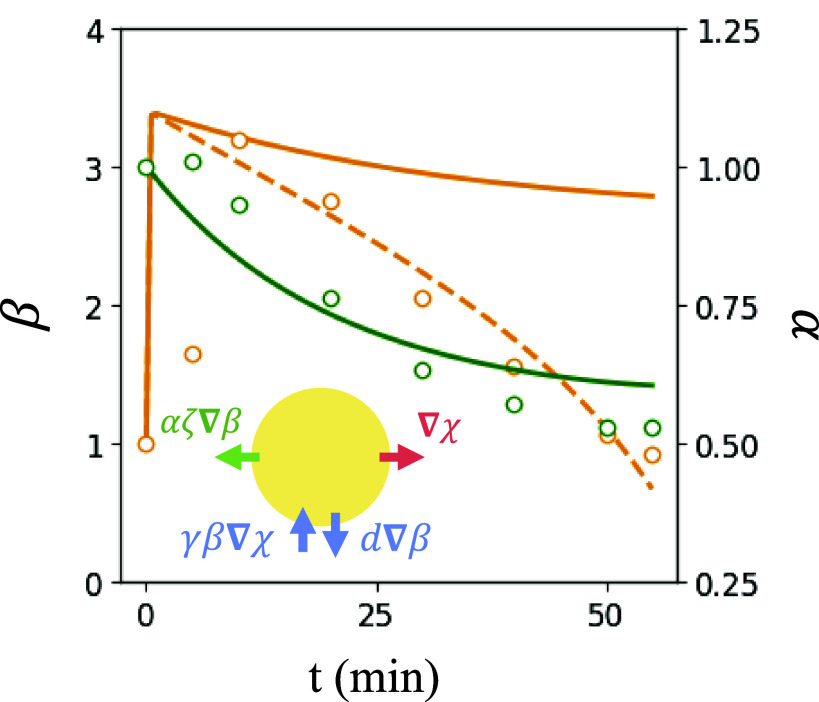
Spatially averaged dynamics. Average concentration of bacteria (orange) and algae (green) in illuminated region from experiments (circles) and model (lines) shown in scattered points. For bacteria, simple chemotaxis is shown by solid orange line, adaptive chemotaxis shown dashed. *Inset*: Schematic of fluxes across the illuminated region.

Insight into the basic process of algal expulsion can be obtained by constructing a spatially averaged model which takes as dynamical degrees of freedom the mean concentrations of algae, bacteria, and oxygen inside the illuminated region, denoted by $\overset{¯}{\alpha }$, $\overset{¯}{\beta }$, and $\overset{¯}{\chi }$. As indicated schematically in Fig. 6, the specification of this averaged model requires estimates of the various fluxes across the boundary of the illuminated region. We anticipate that stochastic bacterial dynamics will homogenize oxygen inside the illuminated region, save for the area close to the boundary of width *λ*, where the concentration rapidly decreases. From an integral form of Eq. **1** and the divergence theorem, we have $\partial_{t}\left(\pi R^{2}\overset{¯}{c}\right)=-D_{c}\oint ds\hat{n}\cdot \nabla c$, where $\hat{n}$ is the outward normal to the illuminated region. If we estimate $\hat{n}\cdot \nabla c∼\overset{¯}{c}/\lambda$, then the PDE Eq. **1** becomes the ODE

[10] $$\frac{d\overset{¯}{\chi }}{\mathrm{dT}}=-2\kappa \overset{¯}{\chi }+\left(1-k\right)\kappa^{2}\overset{¯}{\alpha }-\kappa^{2}\overset{¯}{\beta }\frac{\overset{¯}{\chi }}{1+\epsilon \overset{¯}{\chi }}.$$

Similar estimates hold for the averaged bacteria dynamics, where the relevant fluxes are the outward diffusive and inward chemotactic contributions. The diffusive contribution is precisely analogous to that for oxygen above. Since on the time scales of the experiment only bacteria within a distance *λ* outside the illuminated region sense the gradient, the inward Keller–Segel flux is also estimated as above, but with an upper limit on the bacterial concentration due to steric effects. This leads to the ODE version of Eq. **5**,

[11] $$\frac{d\overset{¯}{\beta }}{\mathrm{dT}}=-2d\kappa \overset{¯}{\beta }+2\gamma \kappa \left(\beta_{\max}-\overset{¯}{\beta }\right).$$

Finally, the algae have an active flux −pa∇b that leads to expulsion, but there is little backdiffusion into the illuminated region. Yet, as algae accumulate at the boundary (Fig. 2), they form a thick ring that inhibits continued expulsion of algae. Introducing a saturation of the algal flux we obtain the ODE version of Eq. **7a**,

[12] $$\frac{d\overset{¯}{\alpha }}{\mathrm{dT}}=2\zeta \kappa \overset{¯}{\beta }\left(\alpha_{\max}-\overset{¯}{\alpha }\right).$$

Using parameter values taken from Fig. 5 and fitting $\beta_{\text{max}}\approx 5$ and $\alpha_{\text{max}}\approx 0.5$, we obtain the solid green and orange curves shown in Fig. 6 for mean algal and bacterial concentrations in the illuminated region. The reduced model captures the slow exponential decay of the algal concentration, but while it reproduces the rapid bacterial influx in the first few minutes of the experiments, the predicted decay of the bacterial concentration, as remarked earlier for the full ABC model, is far slower than observations. The possibility that this discrepancy arises from adaptation of the bacteria to elevated oxygen levels can be explored by assuming, on the time scales of the experiment, a simple linear decay of the chemotactic coefficient, as $\gamma \left(T\right)=\gamma_{0}-T/\tau_{a}$, where *τ*_*a*_ sets the adaptation rate. For $\tau_{a}=1,200$, the bacterial dynamics (dashed orange line in Fig. 6) matches closely with data, while the algal expulsion (not depicted) is essentially unchanged from the nonadaptive case.

## Discussion

The results described here highlight the rich dynamical behavior that occurs in mixed active matter systems involving microorganisms from two Kingdoms of Life. While the dynamics of symbiosis is generally studied on time scales relevant to population dynamics or evolution (40–42), our findings indicate that short-term dynamics on the scale of minutes and hours can have a considerable impact on the spatial-temporal aspects of association, where chemotaxis and phenotypic switching dominate. Although we considered the simple case of light intensity that is piecewise constant, inhomogeneous activity arises. As noted in the Introduction, nonuniform active matter emerges in multiple contexts, including as well the phenomenon of active matter “invasion” (43) and in active gels (44), and is likely important in realistic ecologies. In this sense, generalizing the present setup to allow algal motility and phototaxis in the presence of an inhomogeneous light field may reveal even more striking dynamics when the oxygen sources are themselves motile. Moreover, in the presence of uniform illumination, these models may display pattern-forming instabilities analogous to those in the Keller–Segel model (35). Returning to the motivational problem of bacterial-algal symbiosis, we anticipate even richer dynamics when mutualism is present.

The additional dynamical term that we proposed within the ABC/ABCD models to describe expulsion of algae due to an inhomogeneous bacterial concentration has an interesting similarity to models of magnetic field transport in fluids; a short digression on this connection suggests future areas of study within active matter. The problem of interest is the spatiotemporal dynamics of the magnetic field B within a fluid of velocity u and magnetic diffusivity *D*_*m*_, as described by the induction equation $\partial B/\partial t=\nabla \times \left(u\times B\right)+D_{m}\nabla^{2}B$. Focusing on purely two-dimensional problems confined to the *xy*-plane, the scalar magnitude *A* of the magnetic vector potential $A=A\hat{z}$ obeys the advection–diffusion equation

[13] $$\frac{\partial A}{\partial t}+u\cdot \nabla A=D_{m}\nabla^{2}A.$$

An important problem in MHD has been to understand the dynamics of magnetic fields when u is turbulent, typically by averaging over small-scale features to arrive at effective transport coefficients on larger length scales (32). The result is captured by an equation of motion for the vector potential component $\overset{¯}{A}$ averaged on those scales of the form (45–47)

[14] $$\frac{\partial \overset{¯}{A}}{\partial t}=\nabla \cdot \left(D^{*}\nabla \overset{¯}{A}\right),$$

where $D^{*}$ is a turbulent diffusivity tensor. For the case in which the turbulence varies along one direction, say *y*, then the averaged *x*-component of the magnetic field $F={\overset{¯}{B}}_{x}$ obeys $\partial F/\partial t=\nabla^{2}\left(D^{*}\left(y\right)F\right)$, or

[15] $$\frac{\partial F}{\partial t}-2\left(\partial_{y}D^{*}\right)\partial_{y}F=D^{*}\nabla^{2}F+F\partial_{\mathrm{yy}}D^{*}.$$

With the advective term on the left-hand-side and the reactive term $F\partial_{\mathrm{yy}}D^{*}$ on the right-hand side, we see that the result Eq. **15** has the same structure as our Eq. **7b**, with the bacterial concentration *β* playing the role of the diffusivity tensor component. Thus, the pumping of magnetic fields to regions of lower turbulent activity is analogous to the transport of algae to regions of lower bacterial concentration.

In our experiments, the bacterial concentrations that lead to algal expulsion are below those necessary to support active turbulence (11, 48), yet nevertheless are described by a model whose mathematical structure resembles that in turbulent MHD. An obvious next step is to examine experimentally the transport of passive scalars in inhomogeneous active turbulence as a direct parallel to turbulent pumping in MHD.

## Materials and Methods

The coculture used strain 168 of *B. subtilis* which was genetically engineered to express yellow fluorescent protein m-Venus with excitation at 515 nm and emission at 528 nm (49). A single bacterial colony was picked from an agar plate and grown overnight in Terrific Broth (TB) on an orbital shaker at 240 rpm and 30^°^C. The bacteria intended for experiments were grown from overnight culture until exponential growth phase in Tris-min medium spiked with TB medium (Tris-min + 0.1 % w/v glycerol + 5 % w/v TB). The nonmotile *C. reinhardtii* strain CC477 (*bld1-1*) was sourced from the Chlamydomonas Resource Center (50) and grown in Tris-min medium, on an orbital shaker at 240 rpm and 20^°^C. The diurnal cycle was 12 h cool white light (∼ 15 μmol photons/m^2^s PAR), and 12 h in the dark.

Prior to experiments, bacteria and alga from their respective exponential growth phases were mixed in a modified Tris-min medium, with glycerol as a carbon source and bovine serum albumin to prevent cell adhesion (Tris-min + 0.1% w/v glycerol + 0.01% v/v BSA). The desired number density of cells was achieved by centrifugation at 4,000×g. All cell concentrations and sizes were measured using a Beckman Coulter Counter (Multisizer 4e). The mixture of cells for each experiment was transferred to a glass cover slip chamber with a depth of 300 *μ*m, separated by double-sided tape, and sealed airtight using UV glue. The chamber surfaces were passivated with PEG ($M_{w}=5,000$ g/mol).

Experiments were performed on a Nikon TE2000-U inverted microscope. The spatiotemporal variation in bacterial concentration was monitored by epifluorescence illumination with a ×20 objective using a highly sensitive, backilluminated camera (Teledyne Prime Σ 95B). Movies of algae cells were recorded through the brightfield channel using a Phantom V311 high-speed camera (Vision Research) at ×20 magnification. The halogen lamp served as both a brightfield and photosynthetic light source. The size of a light shaft used to trigger photosynthesis was controlled by the field iris in the microscope condenser arm, producing an octogonal boundary in the focal plane with mean radius of R=220 μm. See *SI Appendix* for more experimental details.

## Supplementary Material

Appendix 01 (PDF)

Movie S1. Movie illustrating Type I algal expulsion. Fluorescence and brightfield video of bacteria and algal dynamics under continuous illumination by a shaft of photosynthetic light extending from the centre of the images up to 440 *μ*m. The concentration of algae was 5 × 10^6^ cm^−3^ while that of bacteria was 1 × 10^8^ cm^−3^.

Movie S2. Movie illustrating Type II algal expulsion. Fluorescence and brightfield video of bacteria and algal dynamics under continuous illumination by a shaft of photosynthetic light extending from the centre of the images up to 440 *μ*m. The concentration of algae was 5 × 10^6^ cm^−3^ while that of bacteria was 5 × 10^8^ cm^−3^.

Movie S3. Movie showing Type I expulsion of algae. Segmented video shows algae being expelled radially outward from the illuminated region as time progresses.

## Data Availability

Experimental results and code have been deposited in Zenodo (51).
