# Supplementary material for: Dynamics of an algae–bacteria microcosm: Photosynthesis, chemotaxis, and expulsion in inhomogeneous active matter
Source: Proc Natl Acad Sci U S A. 2025 Mar 17;122(12):e2410225122. doi: 10.1073/pnas.2410225122 (PMC11962504; doi:10.1073/pnas.2410225122)
Supplement: Supplementary file 1 — Appendix 01 (PDF) [file pnas.2410225122.sapp.pdf]

Supporting Information for

Dynamics of an Algae-Bacteria Microcosm: Photosynthesis, Chemotaxis, and Expulsion in Inhomogeneous Active Matter

Praneet Prakash, Yasa Baig, François J. Peaudecerf and Raymond E. Goldstein

Corresponding Author: Raymond E. Goldstein.
E-mail: R.E.Goldstein@damtp.cam.ac.uk

This PDF file includes:

Supporting text
Figs. S1 to S4
Tables S1 to S3
Legends for Movies S1 to S3
SI References

Other supporting materials for this manuscript include the following:

Movies S1 to S3

Supporting Information Text

1. Overview of Experimental Design

Light illumination and cell phototoxicity. The algae-bacteria mixture was illuminated by a shaft of visible light ($R = 220\,\mu m$) from a broadband halogen lamp (OSRAM 64625 HLX - 100W 12V) that served both as a brightfield and photosynthetic light source. Before every experiment, the power of illuminated light within the octagonal light shaft was measured using a microscope slide power sensor (Thorlabs S170C) and maintained at $20\,\mu$W. This power is lower than the power in the diurnal chamber used for growing algae, where the algae are continuously illuminated for nearly 12 h during their growth phase. In comparison, the total experimental time is just 1 h, and thus algae cells remain completely viable.

Imaging. The halogen lamp served as both a brightfield and photosynthetic light source. For imaging micro-algae, the dichroic is disengaged and iris is momentarily opened to capture the algae distribution beyond the illuminated zone. For fluorescent imaging, the excitation light is briefly activated while the brightfield light is simultaneously turned off, with the dichroic mirror engaged in the light path.

Temperature control. All experiments were carried out in a temperature controlled environment maintained at 20°C. We placed the glass coverslip chamber on a circular stage, allowing maximal metallic contact around the illuminated region. The temperature was monitored for several hours using a 10kΩ NTC thermistor (sensitivity 0.1°C) affixed to the back of the device in the light path, with no change in temperature recorded. The illuminated volume is just $5\,\mu$L, compared to the total volume of upwards of $70\,\mu$L, facilitating the effective dissipation of heat from absorbed light. Additionally, a low cell volume fraction $\phi = 0.001$ allows nearly all light to pass through.

Availability of macronutrients (C, N, P). It is important to maintain the availability of macronutrients (N, P) during the experiment. The preferred medium for algae growth is Tris-min medium (1) and for the bacteria motility is Ordal's chemotaxis medium for *B. subtilis* (2), which is known to support bacterial motility for several hours. Tables S1 and S2 show the composition of Tris minimal medium and Ordal's chemotaxis medium for easy comparison; note that Tris minimal contains all the components of Ordal's chemotaxis medium. We use Tris minimal medium, with glycerol as a carbon source for our experiments (Tris-min + 0.1% w/v glycerol + 0.01% v/v BSA). This medium is preferred since it supports normal metabolism of algae and sustains bacterial motility for several hours, but lacks amino acids necessary for bacterial cell division. Thus, neither of the two type of cells, algae or bacteria divides during the experimental timescale. Before each experiment, the cells are introduced into the fresh medium where the number of algae cells is $\sim 5 \times 10^6\,\mathrm{cm}^{-3}$, which is much lower than the maximum number it can support.

Photosynthesis of *C. reinhardtii* and respiration of *B. subtilis*. The photosynthesis of organic carbohydrates by algae can be summarized (3) by the following reaction:

$$\mathrm{CO_2 + H_2O \xrightarrow{\text{light}} CH_2O + O_2} \qquad [1]$$

This is a simplistic representation and hides several other cellular processes (4). The typical consumption rate of CO_2 is $1 \times 10^{-7}\,\mu\mathrm{mol\,cells^{-1}\,h^{-1}}$ (1.7×10^7 molecules $\mathrm{cells^{-1}\,s^{-1}}$) and the production rate is O_2 is $0.5 - 1 \times 10^{-7}\,\mu\mathrm{mol\,cells^{-1}\,h^{-1}}$ ($0.85 - 1.7 \times 10^7$ molecules $\mathrm{cells^{-1}\,s^{-1}}$) (5–8). Similarly, *B. subtilis* consumes O_2 and releases a proportional amount of CO_2. The uptake rate of O_2 is of the order of $5 \times 10^{-9}\,\mu\mathrm{mol\,cells^{-1}\,h^{-1}}$ (8.5×10^5 molecules $\mathrm{cells^{-1}\,s^{-1}}$) (9, 10). Therefore, in a mixture of light illuminated algae-bacteria, a competition exists between the production and consumption of O_2 and CO_2.

Algal activity without bacteria. The average size of algae cells is $13\,\mu$m with a thermal diffusion coefficient $0.03\,\mu\mathrm{m}^2$/s. With such a low diffusivity, in the absence of bacteria the algae are essentially stationary on the time scale of the experiments, as shown in Fig. S1.

Bacterial activity without algae. When bacteria are introduced into the experimental chamber without algae, their speed at low cell concentrations ($\sim 1 \times 10^6\,\mathrm{cm}^{-3}$) remains nearly constant ($18 - 20\,\mu$m/s) during the experimental timescale (30 min). At higher concentrations, their activity eventually drops due to consumption of O_2. For $b \sim 1 \times 10^8\,\mathrm{cm}^{-3}$, it takes 20-25 minutes for motility to cease, while at $\sim 5 \times 10^8\,\mathrm{cm}^{-3}$ cells become immobile within 5 min.

2. Bacterial Influx and Algal Expulsion

Type I and Type II dynamics: O_2 and CO_2 production and consumption. In our experiments demonstrating Type I and Type II behavior, we maintained the algal concentration at $\sim 5 \times 10^6\,\mathrm{cm}^{-3}$ and bacteria concentration at $\sim 1 \times 10^8\,\mathrm{cm}^{-3}$ for Type I and $\sim 5 \times 10^8\,\mathrm{cm}^{-3}$ for Type II dynamics. Slight variations in the exact number of cells across different repeats (Figs. S2 & S3) are due to the nature of centrifugation and mixing. At lower bacteria concentrations $\sim 5 \times 10^7\,\mathrm{cm}^{-3}$, the influx of bacteria into the illuminated region is very weak and does not lead to algal expulsion within the experimental timescale. While at the higher concentration of $\sim 1 \times 10^9\,\mathrm{cm}^{-3}$, ambient O_2 levels become insufficient to sustain bacterial motility.

From direct experimental observation, we have seen that bacterial motility without algae ceases within 20 mins for $b \sim 1 \times 10^8\,\mathrm{cm}^{-3}$ and within 5 mins for $b \sim 5 \times 10^8\,\mathrm{cm}^{-3}$. Clearly, oxygen is a limiting factor and as explained in Section 1 above, the O_2 production rate of *C. reinhardtii* is 100 times the consumption by *B. subtilis* on a per cell basis. Thus, if

the number of bacteria is nearly 100 times that of algae, a competition between production and consumption of O_2 will be established. This is observed in Type I ($b/a = 50$) and Type II ($b/a = 100$) dynamics.

We note that the dissolved concentration of CO_2 in water is 38 mM (11) in the presence of only CO_2 at 760 mm Hg and 20°C. The dissolve concentration of gases in water is proportional to their partial pressure. Since CO_2 is 0.04% of atmospheric air, as a first approximation we estimate the number of molecules is ($\sim 1 \times 10^{13}$ molecules) near the illuminated region. Here, CO_2 diffusion coefficient is $\sim 10^{-9}$ m^{2}s^{-1} corresponding to a lengthscale of (1 mm) in $\sim 10^3$ s. There are ~ 300 cells in the illuminated region and they consume at most $\sim 3 \times 10^{-11}$ mole ($\sim 1.8 \times 10^{12}$ molecules) during the experimental timescale of 1 hr (5–8). Further, over time bacteria metabolism will lead to even higher CO_2 availability.

A smooth transition in extent of bacterial and algal expulsion with increasing bacterial concentration.. See Fig. S4.

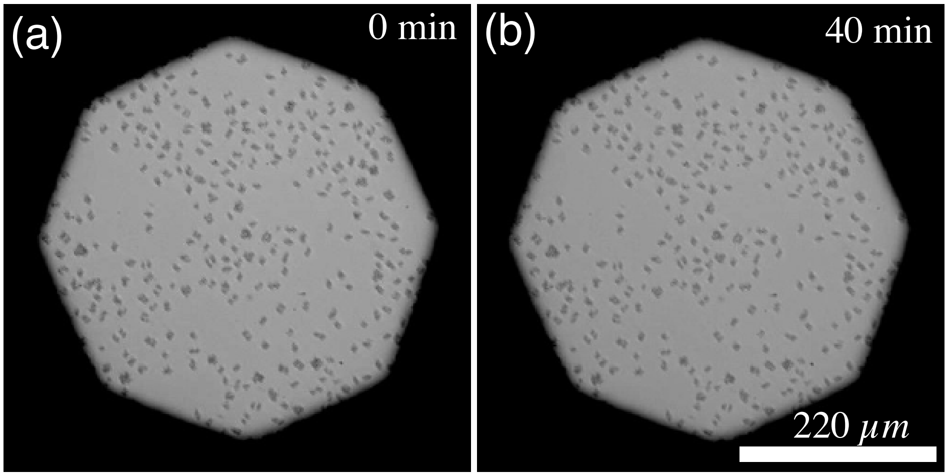

Fig. S1. Test for the effects of light on the algal distribution. (a) Initial distribution ($a = 4 \times 10^6\ \mathrm{cm}^{-3}$). (b) After 40 minutes of continuous illumination there is no discernable change in the spatial distribution.

Table S1. Tris minimal medium

Species	Na^{+}	K^{+}	Mg^{2+}	Ca^{2+}	SO_4^{2-}	NH_4^{+}	PO_4^{3-}	$EDTA^{4-}$
Molarity	312 μM	1.72 mM	410 μM	340 μM	0.41 mM	7.5 mM	1 mM	150 μM

Species	$H_2BO_3^{-}$	Mn^{2+}	Cu^{2+}	MoO_4^{2-}	Co^{2+}	Zn^{2+}	Fe^{2+}	Tris
Molarity	18 μM	76.5 μM	180 μM	25.8 μM	6.4 μM	6.3 μM	6.7 μM	20 mM

Table S2. Ordal's chemotaxis medium

Species	Na^{+}	K^{+}	Ca^{2+}	SO_4^{2-}	NH_4^{+}	PO_4^{3-}	$EDTA^{4-}$	Cl^{-}	lactate
Molarity	5 mM	10 mM	140 μM	300 μM	600 μM	10 mM	100 μM	280 μM	5 mM

Table S3. Values of dimensional and dimensionless parameters associated with the ABC and ABCD models.

Quantity	symbol	value	comments
diffusion constant of oxygen	D_c	2×10^3 μm^2/s	fixed
radius of illuminated region	R	$220\,\mu$m	fixed
derived quantity	ϵ	$k/(1-k)$	fitted
scaled time	T	tD_c/R^2	
scaled radial coordinate	η	r/R	
initial uniform algal concentration	a_0	1×10^6 cm^{-3}	
initial uniform bacterial concentration	b_0	$(1-5) \times 10^8$ cm^{-3}	
scaled algal concentration	α	a/a_0	
scaled oxygen concentration	χ	c/c^*	
scaled bacterial concentration	β	b/b_0	
oxygen consumption time	τ_c	250 s	fixed
scaled dormant bacterial concentration	δ	d/b_0	
screening length	λ	$(D_c\tau_c)^{1/2}$	fixed
oxygen diffusion time	τ_D	R^2/D_c	
length ratio	κ	R/λ	fixed
bacterial diffusion constant	D_b	$200\,\mu$m^2/s	fixed
diffusion constant ratio - bacteria	d	D_b/D_c	fixed
diffusion constant ratio - dormant bacteria	d_d	D_d/D_c	fitted
diffusion constant ratio - algae	d_a	$\tilde{D}_a/D_c$	fitted
algal diffusion parameter	δ^*		fitted
scaled chemotactic parameter	γ	gc^*/D_c	fitted
scaled interconversion rate	ρ	$v_{con}R^2/D$	fitted
scaled interconversion constant	χ^*	K_{sat}/c^*	fitted
scaled advective term - algae	ζ	pb_0/D_c	fitted
scaled advective term - dormant bacteria	ζ_d		fitted
scaled adaptation time	τ_a		fitted

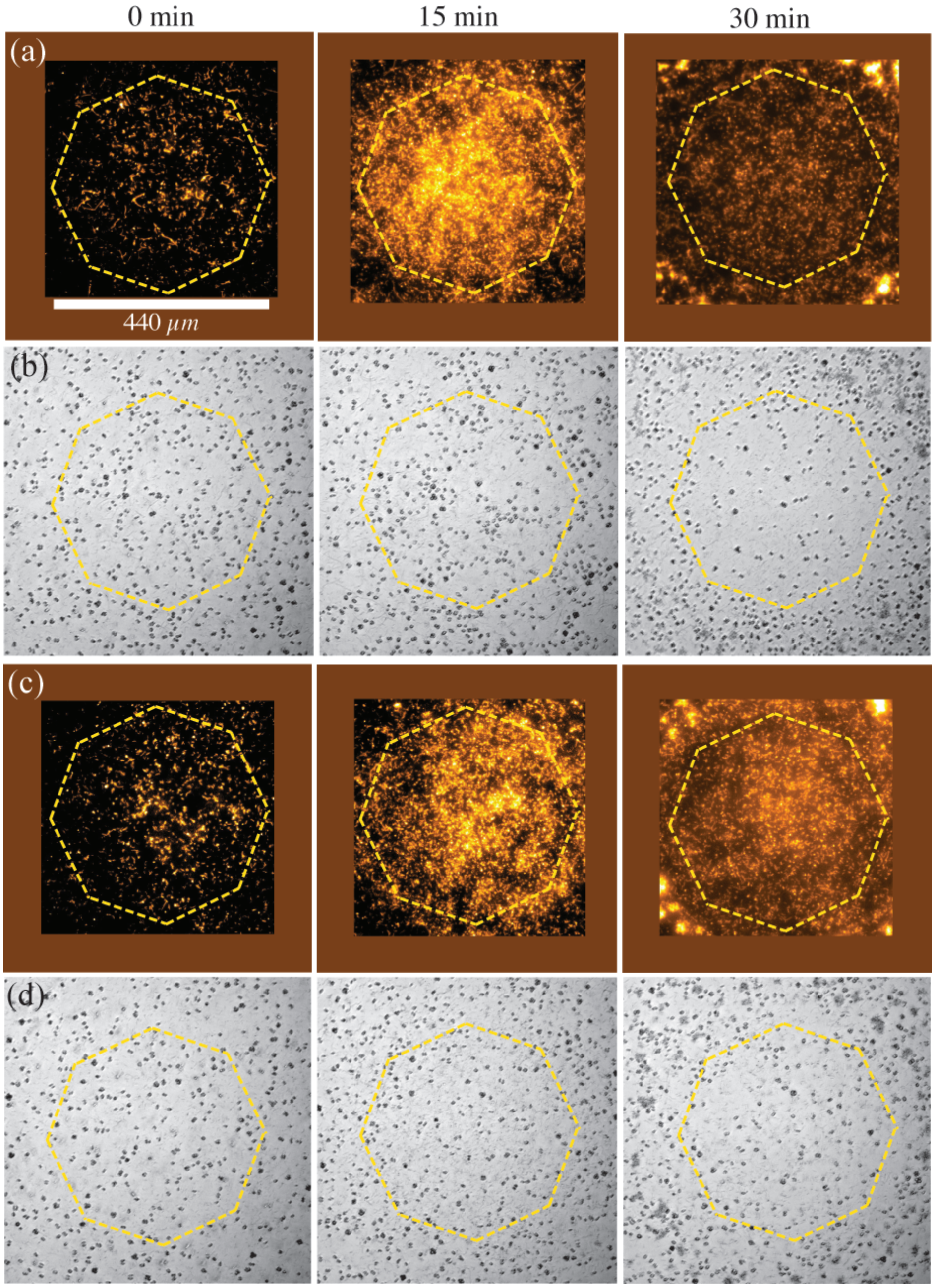

Fig. S2. Repeats of Type I algal expulsion. (a,b) $a = 6 \times 10^6$ cm^{-3}, $b = 1.5 \times 10^8$ cm^{-3}. (c, d) $a = 4 \times 10^6$ cm^{-3}, $b = 1.3 \times 10^8$ cm^{-3}.

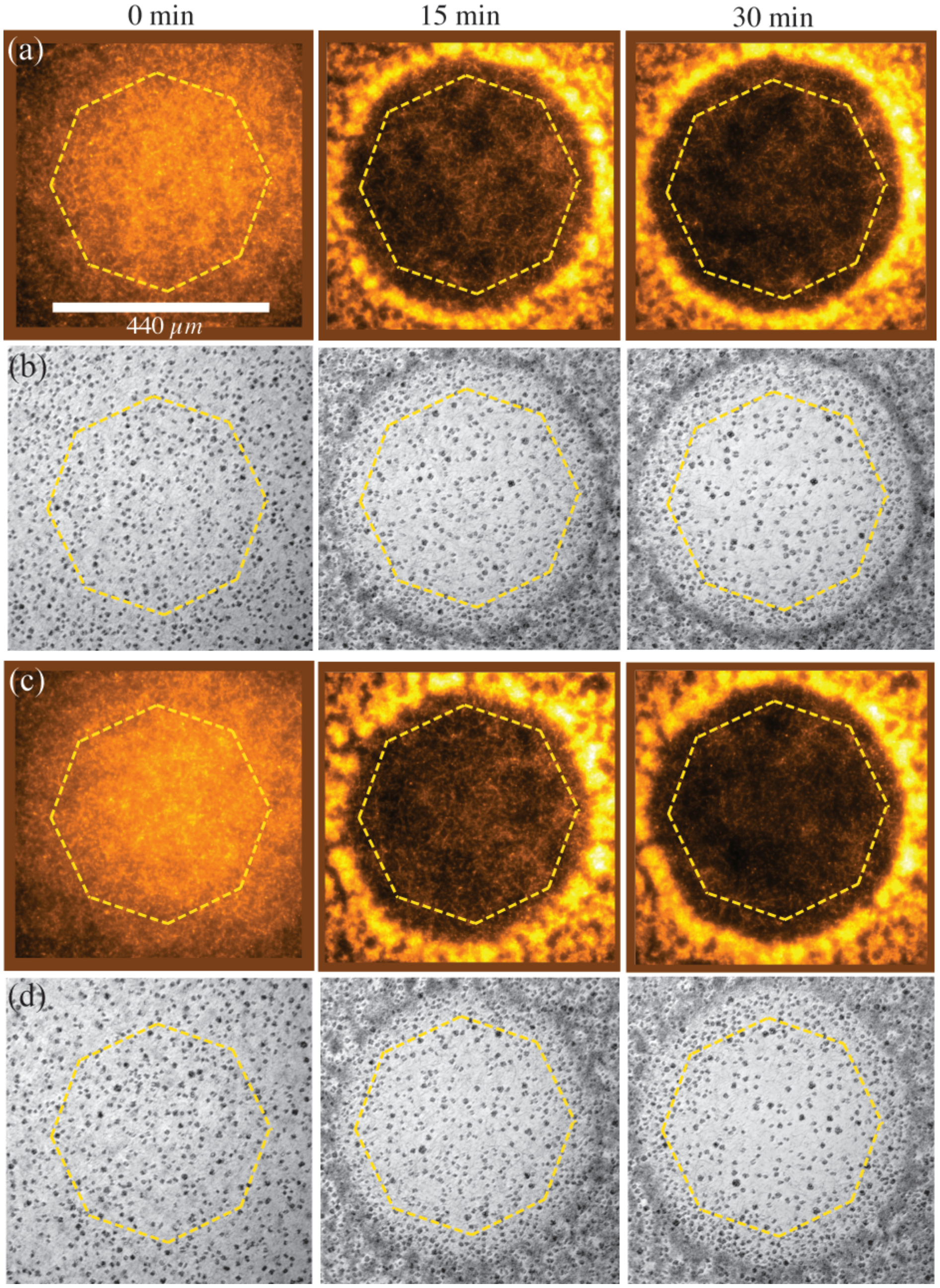

Fig. S3. Repeats of Type II algal expulsion. (a,b) $a = 6.5 \times 10^6$ cm^{-3}, $b = 4.3 \times 10^8$ cm^{-3}. (c, d) $a = 6.5 \times 10^6$ cm^{-3}, $b = 5.0 \times 10^8$ cm^{-3}.

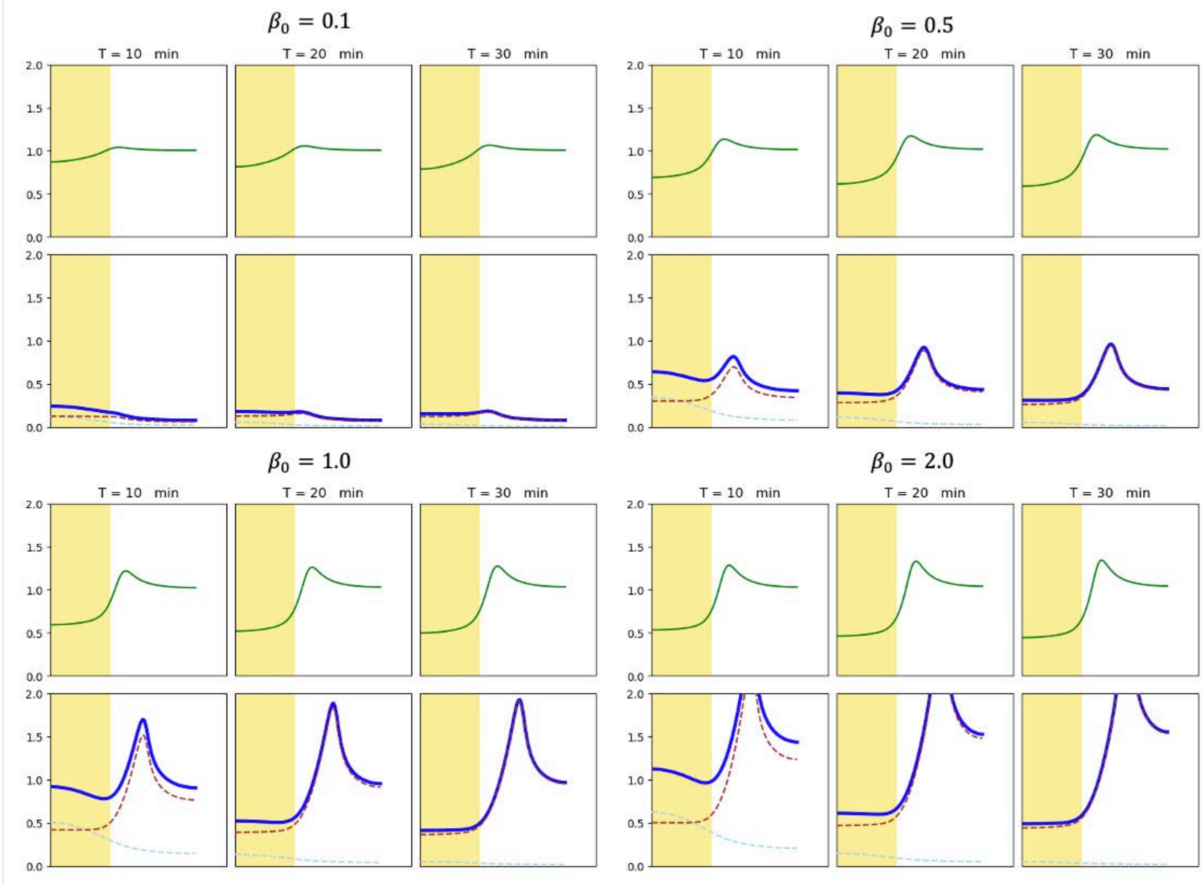

Fig. S4. Effects of varying the initial bacterial concentration on expulsion dynamics. The extent of algal expulsion (green) and dormant bacterial expulsion (brown dashed line) increases smoothly with β_0.

Movie S1. Movie illustrating Type I algal expulsion. Fluorescence and brightfield video of bacteria and algal dynamics under continuous illumination by a shaft of photosynthetic light extending from the centre of the images up to 440 μm. The concentration of algae was 5×10^6 cm^{-3} while that of bacteria was 1×10^8 cm^{-3}.

Movie S2. Movie illustrating Type II algal expulsion. Fluorescence and brightfield video of bacteria and algal dynamics under continuous illumination by a shaft of photosynthetic light extending from the centre of the images up to 440 μm. The concentration of algae was 5×10^6 cm^{-3} while that of bacteria was 5×10^8 cm^{-3}.

Movie S3. Movie showing Type I expulsion of algae. Segmented video shows algae being expelled radially outward from the illuminated region as time progresses.

References

1. https://www.chlamycollection.org/.
2. G.W. Ordal and K.J. Gibson, Chemotaxis toward amino acids by *Bacillus subtilis*, J. Bacteriol. **129**, 151–155 (1977).
3. *The Chlamydomonas Source Book: Organellar and Metabolic Processes*, Vol. 2, A.R. Grossman and F.-A. Wollman, eds. (Academic Press, 2023).
4. M.R. Badger, Photosynthetic Oxygen Exchange, Annu. Rev. Plant Physiol. **36**, 27–53 (1985).
5. P. Vance and M.H. Spalding, Growth, photosynthesis, and gene expression in *Chlamydomonas* over a range of CO_2 concentrations and CO_2/O_2 ratios: CO_2 regulates multiple acclimation states, Canad. J. Bot. **83**, 796–809 (2005).
6. D. Sultmeyer, K. Klug and H. Flock, Effect of dissolved inorganic carbon on oxygen evolution and uptake by *Chlamydomonas reinhardtii* suspensions adapted to ambient and CO_2-enriched air, Photosynth. Res. **12**, 25–33 (1987).
7. G. Peltier and P. Thibault, Light-Dependent Oxygen Uptake, Glycolate, and Ammonia Release in L-Methionine Sulfoximine-Treated *Chlamydomonas*, Plant Physiol. **77**, 281–284 (1985).
8. A. Kaplan and J.A. Berry, Glycolate Excretion and the Oxygen to Carbon Dioxide Net Exchange Ratio during Photosynthesis in *Chlamydomonas reinhardtii*, Plant Physiol. **67**, 229–232 (1981).
9. L.S. Wong, M.S. Johnson, I.B. Zhulin and B.L. Taylor, Role of methylation in aerotaxis in *Bacillus subtilis*, J. Bacteriol. **177**, 3985–3991 (1995).
10. A.J. Hillesdon, T.J. Pedley and J.O. Kessler, The development of concentration gradients in a suspension of chemotactic bacteria, , 57, 299-344, 1995. Bull. Math. Biol. **57**, 299–344 (1995).
11. J.A. Dean, *Lange's Handbook of Chemistry*, 15th edition (McGraw Hill, 1999).
